# Expression of vascular endothelial growth factor-C and vascular endothelial growth factor receptor-3 in esophageal squamous cell carcinoma

**DOI:** 10.3892/ol.2014.1823

**Published:** 2014-01-24

**Authors:** ITARU OMOTO, MASATAKA MATSUMOTO, HIROSHI OKUMURA, YASUTO UCHIKADO, TETSURO SETOYAMA, YOSHIAKI KITA, TETSUHIRO OWAKI, YUKO KIJIMA, HIROYUKI SHINCHI, SUMIYA ISHIGAMI, SHINICHI UENO, SHOJI NATSUGOE

**Affiliations:** Department of Digestive Surgery, Breast and Thyroid Surgery, Graduate School of Medicine, Kagoshima University, Kagoshima 890-8520, Japan

**Keywords:** vascular endothelial growth factor-C, vascular endothelial growth factor receptor-3, esophageal cancer, microlymphatic vessel density

## Abstract

Lymph node metastasis is one of the most important prognostic factors in esophageal squamous cell carcinoma (ESCC). Vascular endothelial growth factor (VEGF)-C and its receptor, VEGF receptor-3 (VEGFR-3), are key in the process of lymphangiogenesis. The present study immunohistochemically examined the expression of VEGF-C, VEGFR-3 and D2-40 in 119 patients with ESCC, and microlymphatic vessel density (MLVD) was calculated based on D2-40 expression counts. Positive expression of VEGF-C was found to correlate significantly with depth of tumor invasion, lymphatic invasion and lymph node metastasis (P<0.001, P<0.0001 and P<0.0001, respectively). Patients with deeper tumor invasion showed higher positivity of VEGFR-3 expression (P<0.05), while patients with lymph node metastasis showed higher MLVD (P<0.05). When patients were divided into three groups according to the expression of VEGF-C and VEGFR-3, patients with coexpression of VEGF-C and VEGFR-3 exhibited poorer prognosis and higher MLVD. The VEGF-C/VEGFR-3 axis is important in tumor lymphangiogenesis.

## Introduction

Esophageal squamous cell carcinoma (ESCC) is one of the most aggressive types of gastrointestinal cancer, due to the relatively high risk of metastasis even in the early stage. In particular, lymph node metastasis is one of the most important prognostic factors (1). Tumor cells take advantage of the lymphatic vascular system to promote metastasis to the lymph nodes and beyond (2). Tumor-induced lymphangiogenesis promotes metastasis to regional lymph nodes and often represents the first step in tumor dissemination. Lymph node metastasis offers a major prognostic indicator for the progression of types of human cancer. Two members of the vascular endothelial growth factor (VEGF) family, VEGF-C and VEGF-D, reportedly induce not only angiogenesis, but also lymphangiogenesis via VEGF receptor (VEGFR)-2 and VEGFR-3 on lymphatic endothelial cells (3,4). These receptors not only regulate lymphangiogenesis, but also enhance lymphatic metastasis (5). In addition, VEGF-C and VEGFR-3, which together have been proposed as a marker for lymphatic endothelial cells, have recently been reported to be expressed by tumor cells in correlation with the invasion, metastasis and progression of cancer cells (6–8).

Several studies have previously examined the roles of the VEGF-C/VEGFR-3 axis and lymphangiogenesis. Lymphangiogenesis is a key factor in nodal metastasis and a prognostic factor for various carcinomas of the esophagus (9), stomach (10–12), colorectum (13), lung (14), cervix (15,16) and prostate (17,18).

The present study aimed to clarify whether expression of VEGF-C and VEGFR-3 in the tumor cells of ESCC correlates with tumor lymphangiogenesis, lymph node metastasis and other clinicopathological factors. In addition, it was examined whether VEGF-C and VEGFR-3 have potential as targets of molecular therapies.

## Materials and methods

### Patients

In total, 119 patients with ESCC (108 males and 11 females) who underwent curative esophagectomy with lymph node dissection between 1996 and 2003 at the Kagoshima University Hospital (Kagoshima, Japan) were enrolled. Patient ages ranged between 38 and 86 years (mean, 65.3 years). Transthoracic esophagectomy by right and left thoracotomy was performed in 89 (74.8%) and six patients (4.2%), respectively. In addition, transhiatal esophagectomy without thoracotomy was performed in 21 patients (17.6%) and abdominal lower esophagectomy was performed in three patients (3.4%). Three-field lymphadenectomy (cervical, mediastinal and abdominal regions) was performed in 42 patients (35.3%), two-field lymphadenectomy (mediastinal and abdominal regions) in 74 patients (62.2%) and one-field (abdominal region) lymphadenectomy in the remaining three patients. The median number of removed lymph nodes was 42 (range, 5–136) and the number of patients with R0 and R1 resection was 107 and 12, respectively. None of these patients underwent endoscopic mucosal or palliative resection, preoperative chemotherapy or radiotherapy, or exhibited synchronous or metachronous cancer in other organs. Specimens of cancer and non-cancerous adjustment tissues were collected from the patients after informed written consent had been obtained in accordance with the institutional guidelines of the hospital.

Clinicopathological observations were based on the criteria of the TNM classification for esophageal carcinoma of the International Union Against Cancer (19). In total, 29 of the ESCCs were classified as well-differentiated, 68 as moderately differentiated and 22 as poorly differentiated. In addition, 26 of the tumors were located in the upper third of the esophagus, 60 in the middle third and 33 in the lower third. Overall, 40 patients exhibited pT1 tumors, 18 exhibited pT2 tumors and 61 exhibited pT3 tumors. Lymph node metastasis was found in 76 of the 119 patients (63.9%) and lymphatic and venous invasion was identified in 74.8% (89/119) and 66.4% (79/119) of patients, respectively. All the M1 tumors exhibited distant lymph node metastases. Each patient was followed up after discharge with a chest X-ray every 1 to 3 months, computed tomography every 3 to 6 months and ultrasonography every 6 months. Bronchoscopy and endoscopy were performed when necessary. Postoperative follow-up data were available for all patients with a median follow-up period of 39 months (range, 1–137 months). Consequently, 51 patients exhibited relapsed disease in the follow-up period.

### Immunohistochemistry

Once the primary lesions had been fixed in 10% formaldehyde and routinely embedded in paraffin, 3-μm-thick sections were prepared for immunohistochemistry. Sections were deparaffinized in xylene, rehydrated in graded ethanol and incubated in 0.3% H_2_O_2_ solution in methanol for 30 min to block endogenous peroxidases. All sections were autoclaved in 10 mM sodium citrate (pH 6.0) for 10 min and allowed to cool at room temperature. Following washing three times with phosphate-buffered saline for 5 min each, sections were treated with 1% bovine serum albumin (Sigma-Aldrich, St Louis, MO, USA) for 30 min at room temperature.

Sections were incubated overnight at 4°C with the following three antibodies: Mouse anti-VEGF-C monoclonal (1:50; Santa Cruz Biotechnology, Santa Cruz, CA, USA), goat anti-VEGFR-3 polyclonal (1:200; R&D Systems, Wiesbaden, Germany) and mouse anti-D2-40 monoclonal (1:50; Dako, Carpinteria, CA, USA). These reactions were developed using an avidin-biotin immunoperoxidase technique (ABC method). The reaction was visualized using the Vectastain Elite ABC kit and 3,3′-diaminobenzidine solution (Vector Laboratories, Burlingame, CA, USA). Sections were then slightly counterstained with hematoxylin.

Expression of VEGF-C and VEGFR-3 in >30% of the cells examined was considered to represent a positive result (9). Expression of VEGF-C and VEGFR-3 was evaluated in 10 fields of ≥100 cells each using high-power (magnification, ×200) light microscopy (BX50, Olympus, Tokyo, Japan). All immunostained slides were evaluated by two independent observers (I.O. and M.M.).

### Evaluation of microlymphatic vessel density (MLVD)

Vessel count was assessed by light microscopy in areas of tumor containing the highest numbers of capillaries at the invasive edge. Highly vascular areas were identified by scanning tumor sections at low power (magnification, ×40 and ×100; DP71, Olympus). In total, six areas showing the highest degree of neovascularization were identified, vessel count was performed in a ×200 field (x20 objective and ×10 ocular) and the mean count for the six fields was determined as MLVD. As previously described by Weidner *et al*, identification of a vessel lumen was not necessary for a structure to be defined as a vessel (20).

### Statistical analysis

Statistical analysis was performed using JMP^®^ 5.0.1 (SAS Institute Inc., Cary, NC, USA), Student’s t-test, χ^2^ test, Kaplan-Meier method and log-rank test. P<0.05 was considered to indicate a statistically significant difference.

## Results

### Expression of VEGF-C, VEGFR-3 and D2-40 in esophageal carcinoma tissue

**Figure 1:**
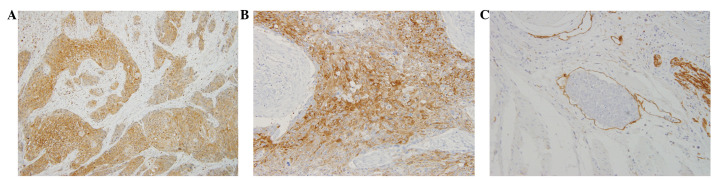
Expression of VEGF-C, VEGFR-3 and D2-40 in esophageal squamous cell carcinoma tissue. (A) VEGF-C (magnification, ×100) and (B) VEGFR-3 (magnification, ×200) were distributed throughout the cytoplasm of cancer cells. (C) D2-40 expression was detected in lymphatic endothelial cells (magnification, ×200). VEGF-C, vascular endothelial growth factor-C; VEGFR-3, vascular endothelial growth factor receptor-3.

Expression of VEGF-C (Fig. 1A) and VEGFR-3 (Fig. 1B) was distributed throughout the cytoplasm of cancer cells. Rates of positive VEGF-C and VEGFR-3 expression were 42.9% (51/119) and 28.6% (34/119), respectively. D2-40 expression was detected in lymphatic endothelial cells (Fig. 1C) and the mean MLVD was 25.8±13.4/field (range, 0–68/field).

### Correlation between clinicopathological factors and expression of VEGF-C and VEGFR-3

Table I shows the correlation between VEGF-C expression and pathological observations. VEGF-C expression was found to correlate significantly with tumor depth, presence of lymph node metastasis and lymphatic invasion (P<0.0001 each). Table I also shows the correlation between VEGFR-3 expression and pathological observations. VEGFR-3 expression was found to correlate significantly with tumor depth and lymphatic invasion (P=0.01 and P=0.032, respectively). Although, the incidence of lymph node metastasis tended to occur in patients with positive expression of VEGFR-3; however, the correlation was not significant.

### Correlation between MLVD and expression of VEGF-C and VEGFR-3

**Figure 2:**
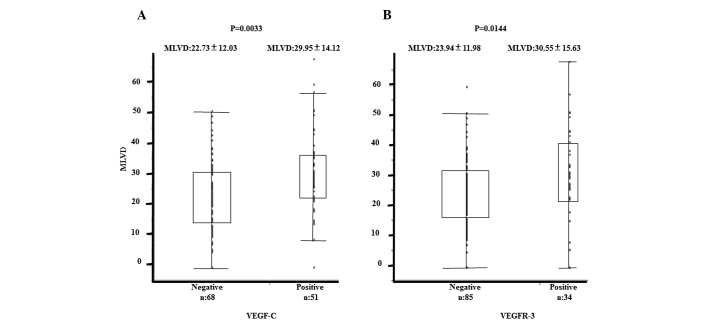
Correlation between MLVD and expression of (A) VEGF-C and (B) VEGFR-3 in esophageal squamous cell carcinoma. MLVD, microlymphatic vessel density; VEGF-C, vascular endothelial growth factor-C; VEGFR-3, vascular endothelial growth factor receptor-3.

Correlations between the expression of VEGF-C and VEGFR-3 and MLVD are shown in Figs. 2A and B. VEGF-C and VEGFR-3 expression was found to correlate significantly with high MLVD (P=0.0033 and P=0.014, respectively). Mean MLVD was 29.95±14.12/field in the VEGF-C-positive group, 22.73±12.03 in the VEGF-C-negative group, 30.55±15.63/field in the VEGFR-3-positive group and 23.94±11.98 in the VEGFR-3-negative group.

### Correlation between prognosis and expression of VEGF-C and VEGFR-3

**Figure 3:**
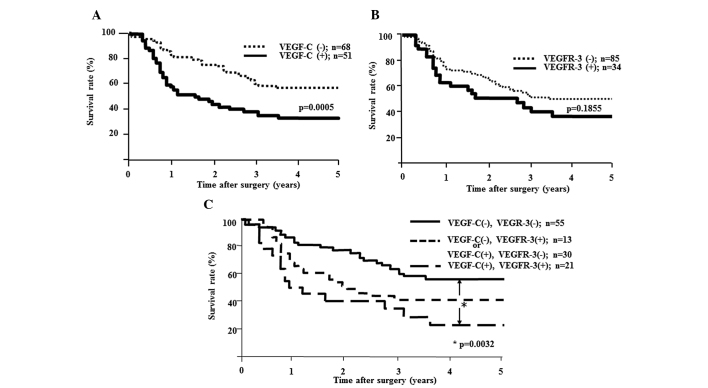
Postoperative survival curves according to (A) VEGF-C, (B) VEGFR-3 and (C) VEGF-C and VEGFR-3 expression. VEGF-C, vascular endothelial growth factor-C; VEGFR-3, vascular endothelial growth factor receptor-3.

Five-year survival rates were analyzed according to the expression of VEGF-C and VEGFR-3. The 5-year survival rate was significantly higher in VEGF-C-negative patients (55%) than in patients with positive expression (31%; P=0.0006; Fig. 3A). No significant difference in 5-year survival rate was found according to the expression of VEGFR-3 (Fig. 3B).

### Prognosis according to the expression of VEGF-C and VEGFR-3

The 5-year survival rate was significantly higher in the double-negative group than in the double-positive group (P=0.0032; Fig. 3C).

### Uni- and multivariate analyses of survival

Univariate analysis showed that the following factors were significantly associated with postoperative survival: Tumor depth, lymph node metastasis, VEGF-C expression, and coexpression of VEGF-C and VEGFR-3 (P<0.05). Multivariate regression analysis indicated depth of tumor invasion and lymph node metastasis as independent prognostic factors (Table II).

## Discussion

Lymphangiogenesis represents an important step in tumor progression and metastasis. Previous studies have revealed that tumors actively induce their own networks of lymphatics that connect with surrounding lymphatic vessels (21–25). The transport of tumor cells by lymphatic vessels represents the most common pathway for initial dissemination, with cancer spread by afferent lymphatics following routes of natural drainage (26–29). Previously, two members of the VEGF family, VEGF-C and VEGF-D, have been associated with lymphangiogenesis and are known as natural ligands for VEGFR-3 (30,31). The present study focused on the expression of VEGF-C and VEGFR-3 and MLVD in ESCC, and evaluated the involvement of the VEGF-C/VEGFR-3 signaling pathway on lymphangiogenesis in ESCC.

In the present study, D2-40 antibody, which reacts with an oncofetal antigen present in fetal germ cells, is a highly reliable lymphatic endothelial marker (32), was first used to detect microlymphatic vessels. Numerous studies have previously indicated that the immunostaining for D2-40 allows specific evaluation of lymphatic invasion and MLVD in types of human cancer (10,33). In the present study, D2-40-expressing microvessels were found in carcinoma tissues, particularly ESCC with lymph node metastases.

With regard to the correlations with clinicopathological features, VEGF-C expression was found to correlate well with several factors, including tumor depth, lymphatic invasion, lymph node metastasis and MLVD, while close correlations with VEGFR-3 expression were limited to tumor depth and MLVD. This may suggest the existence of other pathways for lymphatic spread, but the two molecules were found to closely correlate with each other. These observations suggested that VEGF-C is the most important factor in lymphatic spread and that overexpression of VEGF-C and VEGFR-3 facilitates tumor lymphangiogenesis, resulting in the proliferation of lymphatic vessels. In other words, VEGF-C induces tumor lymphangiogenesis by stimulating VEGFR-3 expression on lymphatic endothelial cells.

Next, the prognosis of ESCC patients was analyzed and patients with overexpression of VEGF-C showed poorer outcomes than those without overexpression, while VEGFR-3 expression was not found to correlate significantly with survival rate. However, expression of VEGF-C and VEGFR-3 resulted in poorer outcomes than other combinations. These results suggested that VEGFR-3 expression in ESCC may have effects only in the presence of sufficient VEGF-C. As previously described in several reports, the VEGF-C/VEGFR-3 axis is critical in cancer progression by inducing lymphangiogenesis and facilitating the mobility of several types of cancer cells. The results of the present study support these previous observations with regard to the role of the VEGF-C/VEGFR-3 axis in the induction of lymphangiogenesis that results in the lymphatic spread of ESCC. MLVD was found to significantly correlate with the VEGF-C/VEGFR-3 system and may present a risk factor for lymph node metastasis and a prognostic factor in ESCC.

Previously, various anti-angiogenic treatments have been applied in clinical situations. VEGF-A and VEGFR-2 are currently the main focus of study. Bevacizumab is a humanized monoclonal antibody against VEGF-A and aflibercept (VEGF-Trap) is a soluble fusion protein for the extracellular domain of VEGFR-1 and VEGFR-2 and the Fc region of immunoglobulin G. These agents neutralize VEGF-A, preventing tumor angiogenesis. VEGFR tyrosine kinase inhibitors, such as sunitinib and sorafenib, are also effective in anti-angiogenic tumor therapy by inhibiting VEGFR signaling. Anti-VEGF drugs currently appear promising as therapies for various cancer patients.

Conversely, lymphangiogenesis shows similar biological mechanisms to angiogenesis. VEGF-C and VEGFR-3 expression, as well as MLVD, may serve as prognostic biomarkers in patients with ESCC (34). Lymphangiogenesis is activated in cancer and inflammation, but is largely inactive in normal physiology, suggesting the therapeutic potential of targeting the underlying mechanisms. As demonstrated in the results of the current study, VEGF-C and VEGFR-3 signaling appear essential for the development of lymphatic vessels and, thus, provide a promising target for the inhibition of tumor lymphangiogenesis. Previously, Burton *et al* (35) emphasized the importance of inhibiting prostate cancer by blockade of the VEGF-C/VEGFR-3 axis. The authors used a VEGF-C ligand trap and antibody directly against VEGFR-3, which significantly reduced tumor lymphangiogenesis and metastasis to regional lymph nodes and distal vital organs without influencing tumor growth.

An additional potential application to clinical situations is the early detection of cancer spread. Previously, Mumprecht *et al* (36) applied immune-positron emission tomography with a lymphatic-specific antibody, LYVE-1, to detect metastases in the early stage. The resulting images suggested the usefulness of this approach in determining the progression of diseases with a marked lymphangiogenic component. In the present study, overexpression of VEGF-C and VEGFR-3 was suggested to induce lymphatic proliferation of the tumor. Obtaining information predictive of lymphatic spread and lymph node metastases must be useful for selecting appropriate strategies for ESCC treatment.

The VEGF-C/VEGFR-3 axis is important in tumor lymphangiogenesis. Targeting the VEGF-C/VEGFR-3 axis may be therapeutically important for cancer metastasis (28,37). The results of the present study may be beneficial for the treatment of patients with ESCC, and new drugs aimed at blocking the VEGF-C/VEGFR-3 axis may be useful for limiting lymph node metastasis. However, several issues remain with regard to the frequency, mechanisms and biological importance of lymphatic metastases. Numerous growth factors appear to be important in determining the lymph node metastatic potential of ESCC. Future study is necessary to clarify the molecular pathways and introduce novel therapeutic options.

## Figures and Tables

**Table I:** Correlation between VEGF-C and VEGFR-3 expression and clinicopathological factors in 119 ESCC patients.

Factors	VEGF-C-positive expression (n=51), n (%)	P-value	VEGFR-3-positive expression (n=34), n (%)	P-value
Histopathological grading		0.4954		0.0859
Grade 1–2 (n=97)	43 (44)		31 (32)	
Grade 3 (n=22)	8 (36)		3 (14)	
Depth of tumor invasion		<0.0001		0.0140
T1 (n=40)	7 (18)		5 (13)	
T2 (n=18)	6 (33)		5 (28)	
T3 (n=61)	38 (62)		24 (39)	
Lymphatic invasion		<0.0001		0.0327
Negative (n=30)	2 (6)		5 (16)	
Positive (n=89)	49 (55)		30 (33)	
Lymph node metastasis		<0.0001		0.3343
Negative (n=43)	6 (14)		10 (23)	
Positive (n=76)	45 (58)		24 (32)	

VEGF-C, vascular endothelial growth factor-C; VEGFR-3, vascular endothelial growth factor receptor-3; ESCC, esophageal squamous cell carcinoma.

**Table II:** Uni- and multivariate analyses of prognostic factors.

Factors	Univariate P-value	Multivariate P-value	95% confidence interval	Hazard ratio
pT1b/pT2-3	<0.0001	0.0017	1.188–2.256	1.610
pN^−/+^	0.0002	0.0095	1.095–2.031	1.473
VEGF-C^−/+^	0.0005	0.1567	0.919–1.649	1.237
VEGF-C^+^, VEGFR-3^+^ and other patterns	0.0210	0.7295	0.760–1.498	0.061

VEGF-C, vascular endothelial growth factor-C; VEGFR-3, vascular endothelial growth factor receptor-3.
